# Functional Trimeric SARS-CoV-2 Envelope Protein Expressed in Stable CHO Cells

**DOI:** 10.3389/fbioe.2021.779359

**Published:** 2021-12-17

**Authors:** Patrick Mayrhofer, Monika Hunjadi, Renate Kunert

**Affiliations:** Department of Biotechnology, Institute of Animal Cell Technology and Systems Biology, University of Natural Resources and Life Sciences (BOKU), Vienna, Austria

**Keywords:** SARS-CoV-2, COVID–19, spike (S) glycoprotein, CR3022, pandemic, envelope protein, perfusion, integrated bioprocessing

## Abstract

The severe acute respiratory syndrome coronavirus 2 (SARS-CoV-2), a β-coronavirus, is the causative agent of the COVID-19 pandemic. One of the three membrane-bound envelope proteins is the spike protein (S), the one responsible for docking to the cellular surface protein ACE2 enabling infection with SARS-CoV-2. Although the structure of the S-protein has distinct similarities to other viral envelope proteins, robust and straightforward protocols for recombinant expression and purification are not described in the literature. Therefore, most studies are done with truncated versions of the protein, like the receptor-binding domain. To learn more about the interaction of the virus with the ACE2 and other cell surface proteins, it is mandatory to provide recombinant spike protein in high structural quality and adequate quantity. Additional mutant variants will give new insights on virus assembly, infection mechanism, and therapeutic drug development. Here, we describe the development of a recombinant CHO cell line stably expressing the extracellular domain of a trimeric variant of the SARS CoV-2 spike protein and discuss significant parameters to be considered during the expression and purification process.

## Introduction

In late 2019, the human population was hit by a novel beta-coronavirus outbreak that challenged the different healthcare systems around the globe. Biotechnological progress of recent decades enabled rapid identification and characterization of the causative agent of this emerging infectious disease and to develop molecular and immunological tests and effective vaccines based on structural features of SARS-CoV-2 (Xiong et al., 2020; Davidson et al., 2021). One key feature of this zoonotic virus is the extracellular domain of the homotrimeric envelop spike protein that mediates attachment and entry into lung epithelial cells upon binding to its receptor, the angiotensin-converting enzyme 2 (ACE2) (Huang et al., 2020; Shang et al., 2020). The spike protein is a highly complex glycoprotein with more than 22 potential N-glycosylation sites, 15 disulfide bonds and forms a homotrimeric structure protruding from the viral surface (Tian et al., 2021). A suitable method for rapid production of proteins for further method development is transient gene expression (TGE). However, mass production using TGE is limited in terms of cheap and efficient gene transfer reagents and high-quality plasmid DNA (Kam et al., 2007; Li et al., 2013; Kirchdoerfer et al., 2018; Chun et al., 2019; Tortorici et al., 2019; Bangaru et al., 2020; Cai et al., 2020; Esposito et al., 2020; Hsieh et al., 2020; Stadlbauer et al., 2020; Xiong et al., 2020; Budge et al., 2021; Castro et al., 2021; Herrera et al., 2021; Schaub et al., 2021). Furthermore, uptake of multiple plasmid copies and extrachromosomal expression in the producer cells lead to cellular stress conditions, and the culture supernatant is usually harvested at suboptimal viability to improve product yield. Consequently, the downstream purification process is more challenging due to the poor product quality and higher amounts of impurities. In contrast, stable cell lines maintain the expression capacity over extended population doubling times and are independent of the culture scale, making them an attractive option for large-scale manufacturing of this complex protein (Allen et al., 2021; Cibelli et al., 2021; Johari et al., 2021). In order to express the spike protein independent from the viral environment, an engineered version of the extracellular part of the S-protein was designed (Genbank QJE37812.1), and the genetic construct was obtained from the laboratory of Prof. Krammer at the Mount Sinai School of Medicine at the beginning of the pandemic (Amanat et al., 2020). In this “chimeric spike solubleˮ (CSs) protein, two mutations were introduced to stabilize the structure of the protein: K986P and V987P at a helix-turn-helix motif to retain the S2 in the prefusion conformation and thereby disfavor the refolding due to restricted backbone torsion angles. Previous studies demonstrated that proline substitutions in the loop between the first heptad repeat (HR1) and the central helix restrict premature triggering of the fusion protein and often increase expression yields of prefusion ectodomains. The rearrangements of class I fusion proteins are a key step in the transition to the post-fusion conformation. In order to prevent the cleavage between S1 and S2 by furin, the polybasic cleavage site was modified (Wrapp et al., 2020), which makes the modified, recombinant SARS CoV-2 spike protein (CSs) also more similar to the SARS-CoV and the MERS S-protein. At the C-terminus, a chimeric foldon domain of the T4 phage was added to promote trimerization of the molecule (Meier et al., 2004). These modifications resulted in a stabilized version of the extracellular domain of the SARS CoV-2 S-protein and were designed according to the first reported genome sequence of 2019-nCoV (Wu et al., 2020), positions 1–1,208. The structure of this recombinant S-protein was analyzed in electron microscopy (Wrapp et al., 2020).

We demonstrate a workflow that started early in the pandemic using existing cell culture platforms established in the research group to rapidly and efficiently produce recombinant cell lines of the soluble version of the trimeric spike protein in high yield. Analytical methods to quantify and evaluate the quality of this critical protein are highlighted and were developed early to support the entire cell line development. Finally, highly efficient bioprocesses, based on shake tubes or lab-scale bioreactors cultivated in perfusion and traditional fed-batch mode followed by a primary affinity capture step using immobilized metal affinity chromatography (IMAC), are presented. Final harvest titers could be increased by a factor of 10 using a hypothermic temperature shift in bioreactor fed-batch cultures. The presented IMAC purification elution profile was optimized for purity and quality rather than yield, which is diligently presented in other studies (Johari et al., 2021). The produced spike protein showed characteristic binding to anti-SARS mAb CR3022 and polyclonal immunoglobulins isolated from hyperimmunoglobulin preparations. Furthermore, the spike protein was successfully applied in serological ELISA.

## Materials and methods

### SARS-CoV-2 Chimeric Spike Soluble Protein

The recombinant chimeric spike soluble (CSs) protein (Genbank QJE37812.1, Amanat et al., 2020) comprises the Sars-CoV-2 S1/S2 spike protein with two stabilizing proline mutations (K986P, V987P) and a mutation of the polybasic cleavage site (RRAR) together with a thrombin cleavage site. The chimeric foldon domain of the T4 phage was added at the C-terminus of the protein to promote trimerization of the molecule (Meier et al., 2004) followed by a 6x His tag. The mature CSs monomeric chain, thus, consists of 1,242 amino acids, 30 cysteine residues, and 22 potential N-glycosylation sites. The calculated molecular weight of the monomeric CSs chain is 180 kDa, assuming 2.5 kDa per glycosylation site. Similarly, a theoretical molecular weight of 540 kDa is calculated for the full trimeric CSs spike protein. Amino acid sequence information is presented.

### The Expression Vector and Cloning Strategy

The original plasmid pCAGGS-CSs (Amanat et al., 2020) was received from Prof. Krammer at the Mount Sinai Medical School (New York City, NY, USA) and was co-transfected with pCIneo (Promega) that harbors the G418 selection marker. For stable cell line generation, the open reading frame of CSs was cloned into the AscI-linearized pL mammalian expression vector (Mader et al., 2012; Zboray et al., 2015), resulting in pL-CSs. The backbone of pL includes a chimeric CAGGS promoter (CMV enhancer, chicken beta-actin promoter, chimeric intron of chicken beta-actin and rabbit beta-globin), bovine growth hormone (bGH) poly(A) signal, phosphoglycerate kinase promoter driving expression of the neomycin phosphotransferase gene (NeoR/KanR) for G418 resistance, a bacterial origin of replication (ori), bacterial ampicillin resistance gene (AmpR) and a 5′ and 3′ homology region (5′HR and 3′HR) for potential recombination into bacterial artificial chromosomes (BACs).

A suspension CHO-K1 cell line adapted to ActiPro medium (Cytiva) + 6 mM L-gln was chosen as the preferred host cell line for recombinant protein expression. It was previously demonstrated that this host cell line is particularly suitable for recombinant protein production (Reinhart et al., 2018). Alternatively, these cells can be cultivated in CDCHO medium (Gibco) without negatively impacting cellular growth. For transfection, 10 µg of plasmid DNA was resuspended in 200 µl of CD-DG44 medium (Gibco). Five micrograms of pCAGGS-CSs and 5 µg of pCIneo were mixed and transfected to generate initial transfection pools and early protein production. Similarly, 10 µg of pL-CSs was transfected for the establishment of monoclonal stable high-producing cell lines. Lipofectin, 50 µl, was mixed with 150 µl of CD-DG44, incubated for 35 min, then pooled with the plasmid solution and incubated for 10 min. The Lipoplex solution was then added to 2 × 10^6^ viable CHO-K1 cells in 3.6 ml CD-DG44 + 8 mM stable L-gln + 15 mg/L phenol red and incubated for 24 h at 220 rpm. At 24 h post-transfection, the cells were centrifuged and resuspended in 10 ml of CDCHO + 8 mM stable L-gln + 15 mg/L phenol red. A 2-day post-transfection (dpt) selection was started by centrifugation of the cells and resuspension in 4 ml of CDCHO + 8 mM stable L-gln + 15 mg/L phenol red + 0.5 mg/ml G418 and seeded at 2,500 cells/well in 50 µl in a 384 deep-well plate. The plate was fed with 50 µl of selection medium 10 dpt, and growing wells were expanded to 96-well plates and finally to 50 ml of shake tubes starting at 16 dpt. High-producing subclones were then generated by the second round of single-cell limiting dilution subcloning in 384-well plates to select homogenous monoclonal subclones.

### Immobilized Metal Affinity Chromatography Purification of Chimeric Spike Soluble from Initial Cell Pools

The initial cell pool of pCAGGS-CSs/pCIneo co-transfected CHO-K1 cells was cultivated in 200-ml shake tubes (ST200) at 50-ml working volume in ActiPro supplemented with Cell Boost 1 and 3 (Cytiva) and 8 mM L-glutamine with a daily medium exchange to increase the maximum cell density, called semi-continuous perfusion process. Tangential flow filtration with a MWCO of 100 kDa was used to concentrate the supernatant and exchange the buffer to 20 mM sodium phosphate + 0.5 M NaCl + 40 mM imidazole. A HisTrap Fast Flow (FF) column (Cytiva) was used for IMAC purification and CSs was eluted by 20 mM sodium phosphate + 0.5 M NaCl + 500 mM imidazole. Finally, a PD10 column was used for buffer exchange to PBS, and a 15-kDa Amicon column to concentrate the sample. The protein solution was quantified by total A280 absorbance and frozen to −80°C.

### Bioprocessing

The parental stable CSs clone 131A4 and the final subclone CSs C11 (short “C11”) were cultivated in different bioprocesses and different media. C11 was cultivated in lab-scale fed-batch bioreactors or subjected to an optimized semi-continuous perfusion protocol.

For semi-continuous perfusion cultivation, 50 ml of cell suspension was seeded at 10 × 10^6^ viable cells/ml in a ST200 in CDCHO + 8 mM L-gln and incubated at 220 rpm, 50 mm shaking amplitude, 37°C, humid 7% CO_2_ atmosphere in a Kuhner shaker incubator. Exactly every 24 h, cells were centrifuged at 1,300 rpm for 7 min, the supernatant was collected and stored at 4°C, and the cell pellet was disrupted and resuspended in fresh pre-warmed medium. An exact protocol for semi-continuous perfusion cultivation can be found in Mayrhofer & Kunert (2020).

For the integrated perfusion experiment of clone 131A4, a ReadyToProcess WAVE25 bioreactor (Cytiva) was used with ActiPro medium supplemented with Cell Boost 1 and 3 (Cytiva) and 8 mM L-glutamine. An Äkta Pure system equipped with HisTrap Excel was directly integrated with the perfusion harvest line. Fed-batch cultivation was performed in a 500-ml DASGIP system and CDM4HEK293 basal medium fed with Cell Boost 7a and 7b (Cytiva).

### Immobilized Metal Affinity Chromatography Purification Strategy

The supernatant was concentrated and buffer exchanged to running buffer containing 20 mM phosphate buffer, 150 mM NaCl, 20 mM imidazole, pH 7.4 by TFF using a 100-kDa membrane. Stepwise elution was initiated by 20%, 40%, 70%, and 100% of elution buffer containing 20 mM phosphate buffer, 150 mM NaCl, 500 mM imidazole, pH 7.4. Pooled elution fractions were loaded onto Amicon Ultra-15 centrifugal filter devices with a 100-kDa cutoff, and buffer was exchanged to 20 mM Tris-HCl, 150 mM NaCl, pH 8.0. Protein content was quantified by total A280 absorbance or quantitative CSs-specific ACE2/anti-His sandwich ELISA. Samples were 0.2 µm filtered and snap frozen to −80°C.

### Quantification of Chimeric Spike Soluble Samples, Determination of Affinity and Serological Evaluation

A quantitative ACE2/anti-His sandwich ELISA was developed for the quantification of CSs samples. The 96-well MaxiSorp plates (Thermo Fisher) were coated with 5 μg/ml of ACE2 (R&D systems) in PBS overnight at 4°C or 2 h at room temperature. Unknown samples or 1 μg/ml of purified CSs standard were serially diluted in PBS with 0.1% Tween (TPBS) and 1% BSA. Coated plates were washed with TPBS, and the applied samples were incubated for 1 h at room temperature. Biotinylated anti-His (Thermo Scientific) detection antibody was diluted at 1:1,000 in TPBS + 1% BSA and incubated on the plate for 1 h at room temperature. Streptavidin–HRP conjugate (Roche) was diluted at 1:5,000 in TPBS + 1% BSA and incubated on the plate for 30 min at room temperature. The color reaction was developed using TMB substrate and stopped with concentrated H_2_SO_4_ followed by OD 450-nm measurement at 620-nm reference. The EC50 value of CSs binding to ACE2 was determined by fitting a non-linear four-parametric sigmoidal log model on the absorbance values at different CSs molar concentrations. To evaluate the binding affinity of the anti-SARS monoclonal antibody CR3022, purified CSs protein (5 μg/ml) was coated, and serially diluted biotinylated CR3022 was prepared starting at 2 μg/ml (13 nM) in TPBS + 1% BSA. After incubation for 1 h at room temperature, the detection was performed with streptavidin–HRP conjugate (1:5,000) and TMP substrate.

A similar ELISA setup was used to confirm the CSs protein quality with vaccinated donor samples. CS-coated plates were blocked for 1 h at room temperature with TPBS containing 3% of non-fat skim milk powder (MP). Samples were diluted in TPBS with 3% MP and incubated for 2 h at room temperature. Anti-human gamma-HRP detection antibody (Invitrogen) was diluted at 1:5,000 and incubated for 1 h at room temperature. A color reaction was developed using the TMB substrate.

EC50 values were determined by fitting a non-linear four-parametric sigmoidal log model on the absorbance values at different CSs molar concentrations.

### Isolation and Biotinylation of Polyclonal Anti-SARS-CoV-2 Hyperimmune Ig

SARS-CoV-2 convalescent plasma (Takeda) was thawed and cryoprecipitated at 4°C for 2 h followed by centrifugation at 5,000 × *g* for 15 min. Supernatant, 1 ml, was diluted with 2 ml of PBS, and 2.45 ml of saturated ammonium sulfate was added under continuous stirring, and the mixture was incubated at 4°C for 5 h. After centrifugation at 1,000 × *g* for 15 min, the precipitate was washed with 5 ml of 45% saturated ammonium sulfate solution and centrifuged at 1,000 × *g* for 15 min. The pellet was resuspended in 1 ml PBS and centrifuged again at 1,000 × *g* for 2 min. The supernatant was dialyzed against PBS overnight at 4°C and centrifuged again at 10,000 × *g* for 5 min. Following 0.22-µm filtration and storage at 4°C, 1 ml of cryo- and ammonium sulfate precipitated hyperimmune serum was diluted with 9 ml of phosphate buffer and loaded onto a protein A affinity column. Polyclonal hyperimmune IgGs were eluted with 50 mM sodium acetate pH 3.5 and neutralized with Tris-HCl pH 8.0. Hyperimmune IgG, 1.54 mg, was biotinylated using the EZ-Link NHS-PEG4-Biotin kit (ThermoFisher).

### SDS-PAGE and Western Blotting

Bis-Tris NuPAGE polyacrylamide gels, 4%–12%, were run in MOPS SDS running buffer. Samples were incubated with NuPAGE LDS sample buffer for 10 min at 70°C for non-reducing conditions or additionally mixed with NuPAGE reducing agent and incubated for 5 min at 98°C for reducing conditions. The gels were run at 200 V for approximately 1 h at room temperature. A PVDF membrane was activated for 1 min with methanol (MeOH) and rinsed with NuPAGE transfer buffer in 20% MeOH for Western blotting. Proteins were blotted from the SDS-Gel onto the PVDF membrane using a PerfectBlue “Semi-Dry” Electro Blotter (peqlab) at 10 V, 3 mA/cm^2^ for 1 h. The blotted membrane was blocked with 5% BSA in TPBS for 1 h or overnight and washed with TPBS. Detection was performed for 1 h with primary detection antibody in TPBS with 0.5% BSA. Secondary detection was performed with streptavidin–HRP conjugate in TPBS with 0.5% BSA for 30 min. Chemiluminescence was induced with the SuperSignal West Pico Plus substrate (Thermo Fisher).

## Results and discussion

### SARS-CoV-2 Chimeric Spike Soluble Protein from a G418 Selected Early Transfection Pool

**FIGURE 1:**
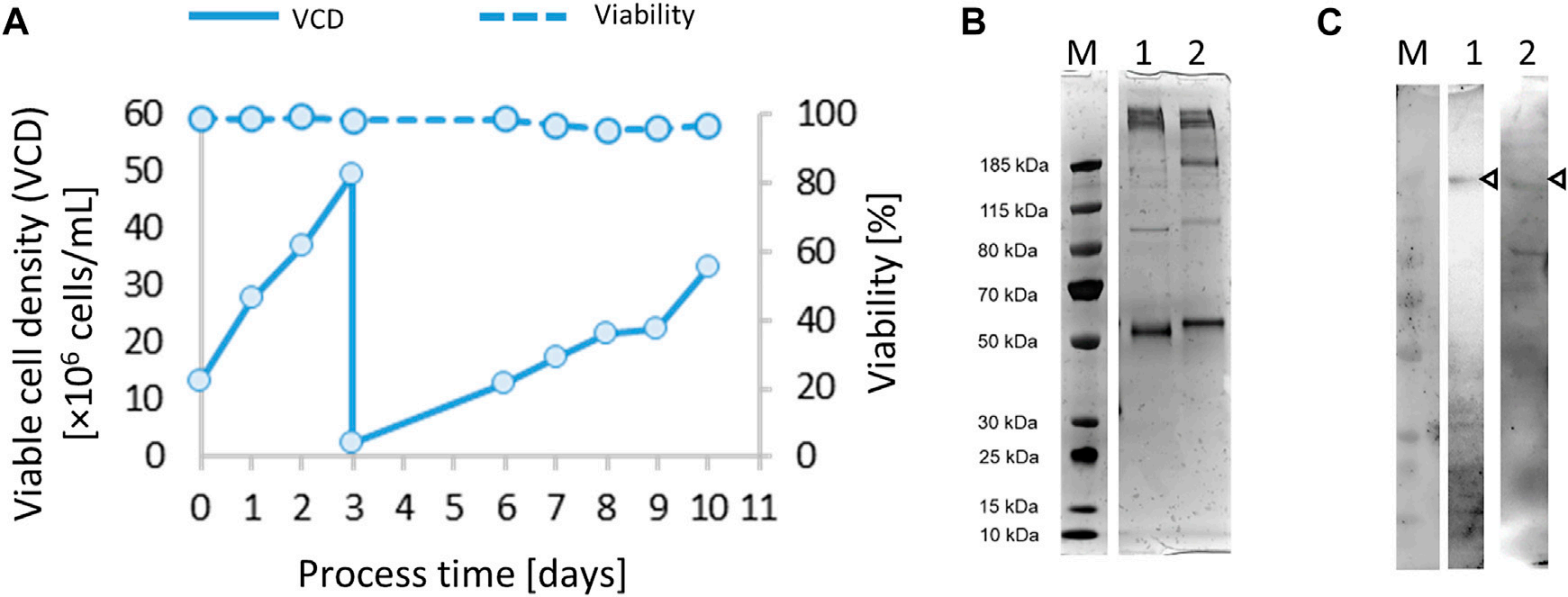
After co-transfection of vectors pCAGGS-chimeric spike soluble (CSs) and pCIneo, and selection of transfection pools for 25 days, a 50-ml semi-continuous perfusion cultivation was started **(A)**. Samples from immobilized metal affinity chromatography (IMAC) (HisTrap) purification were analyzed by 4%–12% Bis-Tris SDS-PAGE (silver stain) **(B)** under non-reducing (lane 1, 500 ng of total protein) and reducing conditions (lane 2, 500 ng of total protein) and Western blotting **(C)** detected with the anti-SARS mAb CR3022/anti-huGamma-HRP (lane 1, 2 µg total protein) or hyperimmune IgG/anti-huGamma-HRP (lane 2, 2 µg total protein).

Preliminary material of a new protein of interest is needed to rapidly develop analytical methods supporting the selection of high-producing subclones during clone development. The originally received plasmid (pCAGGS-CSs) (Amanat et al., 2020) was co-transfected with a vector (pCIneo) containing a suitable G418 selection marker in a quick and straightforward effort. Transfection pools were generated by lipofection, G418 pool selection and after recovery of the viability above 95%, expansion to higher cell concentration using shake tube bioreactors. A semi-continuous perfusion culture starting already 25 days post-transfection was used to generate enough protein to develop analytical methods. Using a daily medium exchange protocol, high cell densities of 50 × 10^6^ viable cells/ml were achieved and passaged once to maintain high viability (Figure 1A). The culture supernatant for optimization of the IMAC purification step was collected from harvests of the semi-continuous perfusion culture of the initial transfection pool.

The purified CSs preparation (Figures 1B, C) was suitable to support essential analytical methods for further clone development (Table 1). The analytical workflow aims to screen, identify, and select high-producing clones. The rapid production of a new recombinant protein such as the SARS CoV-2 spike protein faces several challenges, particularly the availability of suitable reagents (Esposito et al., 2020). One strategy is to use generic analytic methods, e.g., total protein visualization by SDS gel electrophoresis, or ELISA-based methods that detect commonly used protein tags (e.g., 6xHis). However, these methods only provide limited information about the quality of the expressed protein. Thus, several strategies to detect the functional spike protein were pursued in parallel and are summarized in Table 1. Already quite early in the pandemic, the monoclonal antibody CR3022 binding to the receptor binding domain (RBD) of SARS-CoV-2 spike protein was available. This mAb was initially discovered to be specific for SARS CoV (van den Brink et al., 2005) and is commercially available. To be independent of the global market, we also harvested culture supernatants from the early transfection/selection pool and of subclones of recombinant CR3022 CHO-K1 cells and cultivated them in fed-batch bioreactors to yield low but sufficient 10 mg/L of mAb titer at harvest. The ACE2 receptor was commercially available and used for analytical purposes. Using these and other commonly available reagents, various ELISA setups were tested and optimized and the ACE2/anti-His sandwich ELISA gave the best reproducible outcomes.

**TABLE 1:** ELISA and Western blotting repertoire for analytical screening of chimeric spike soluble (CSs) subclones and to verify the quality and functionality of the expressed CSs protein.

Sandwich ELISA	Coating protein	Detection mAb	Comment
	ACE2	Anti-His-HRP or anti-His-btn	High signal used for CSs sample quantification and EC50 quantification (Figure 2 and 8C)
	ACE2	CR3022-btn	Limited CSs detection based on up/down conformation of the RBD-domain (Henderson et al., 2020; Yuan et al., 2020)
	ACE2	Hyperimmune IgG-btn	Low signal (data not shown)
	CR3022	Anti-His-HRP or anti-HIS-btn	Limited CSs detection based on up/down conformation of the RBD-domain (Henderson et al., 2020; Yuan et al., 2020)
	Hyperimmune IgGs	Anti-His-HRP or anti-HIS-btn	low signal (data not shown)
	Purified CSs	Anti-human-gamma-HRP	Serological ELISA (Figure 8E)
**Direct ELISA**	**Coating protein**	**Detection protein**	-
	Purified CSs	CR3022-btn	Strong signal used for EC50 determination (Figure 8D)
	Purified CSs	Hyperimmune IgG-btn	Serological evaluation of CSs (data not shown)
	ACE2	Purified CSs -btn	Not tested. It can be used for binding interaction studies (EC50)
**Western blotting**	**-**	**Detection mAb**	-
		Anti-His-HRP or anti-His-btn	Strong signal (Figure 3C)
		CR3022-btn	Strong signal (Figure 8A)
		Hyperimmune IgG-btn	Strong signal (Figure 7C)

### Recombinant CHO-K1 Cells Stably Expressing SARS-CoV-2 Chimeric Spike Soluble Spike Protein

**FIGURE 2:**
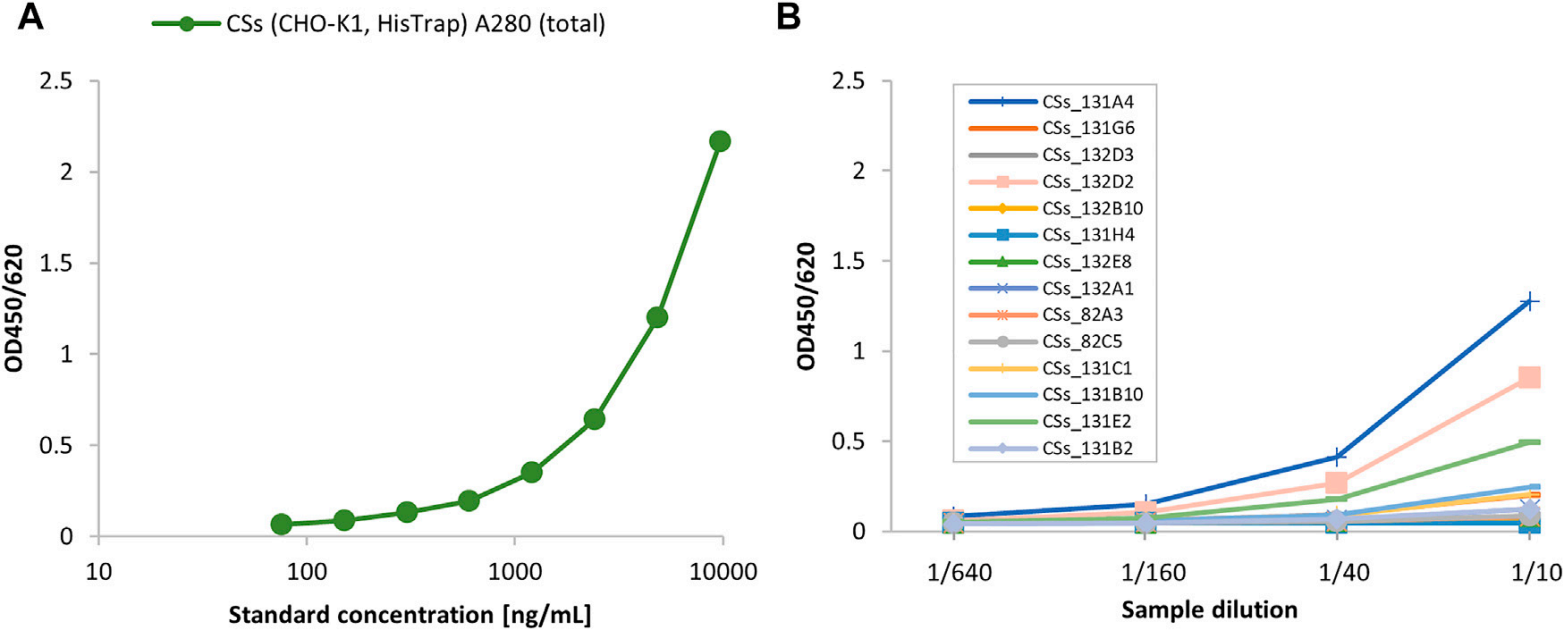
Screening of various parental CSs subclones using the angiotensin-converting enzyme 2 (ACE2)/anti-His sandwich ELISA. The initially purified CSs material from the cell pool cultivation was used as ELISA standard (10 μg/ml total protein by A280 absorbance) **(A)** to screen parental CSs subclones **(B)**.

The pL-CSs construct was used for the generation of recombinant CHO-K1 cell lines stably expressing SARS-CoV-2 spike protein. Purified CSs protein from the initial pool experiment (Figure 1) was used as reference material to select the best producing CSs subclones, as shown in the following ELISA experiments (Figure 2). Parental clone 131A4 reproducibly gave the highest titers and good growth behavior and was selected as the leading parental cell clone.

**FIGURE 3:**
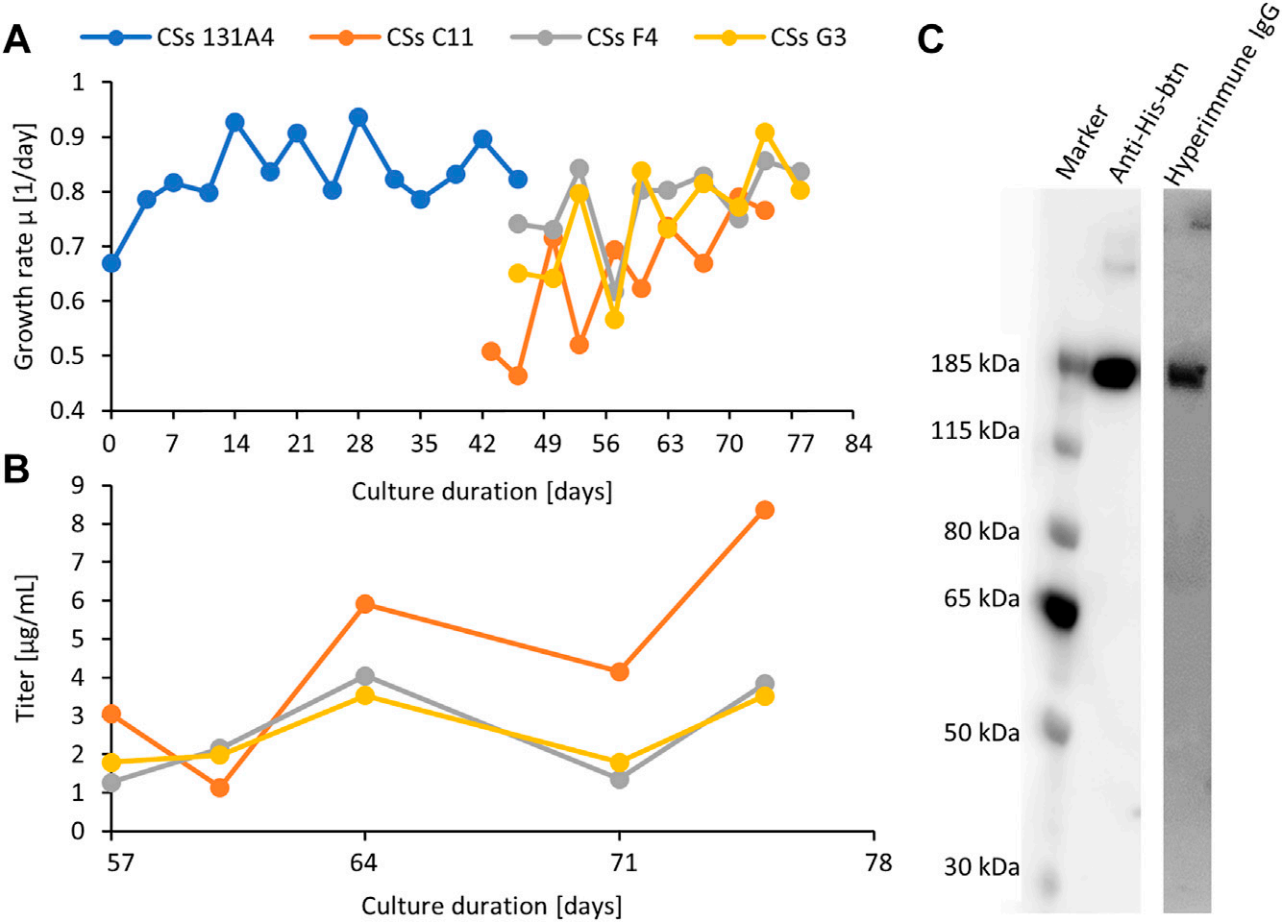
Expansion and monitoring of growth **(A)** and productivity **(B)** of the first parental clone CSs 131A4 and three final CSs daughter clones in shake tube cultures. The clone CSs C11 was selected as the final high-producing subclone. Anti-His and hyperimmune IgG Western blotting **(C)** of C11 supernatant (12 μl, 100 ng) indicate the presence of the recombinant CSs protein in a structurally functional form to bind to specific antibodies. Lane 1, anti-His-btn; lane 2, hyperimmune IgG-btn.

Finally, the parental clone 131A4 was selected for limited dilution subcloning. Three subclones were selected by ELISA quantification of cell culture supernatants and further expanded into shake cultures to characterize growth behavior and productivity (Figure 3). Subclone C11 showed high growth rates of 0.8 days^−1^ comparable with the parental clone and highest titers of 6 mg/L after 3 to 4 days of routine cultivation (Figures 3A, B). All clones showed the correct mass of the monomeric spike protein in Western blotting under reducing and denaturing conditions detected with anti-His or hyperimmune IgGs (Figure 3C).

### Scale-up of Spike Protein Expression by Perfusion Cultivation and Hypothermic Fed-Batch Cultivation

**FIGURE 4:**
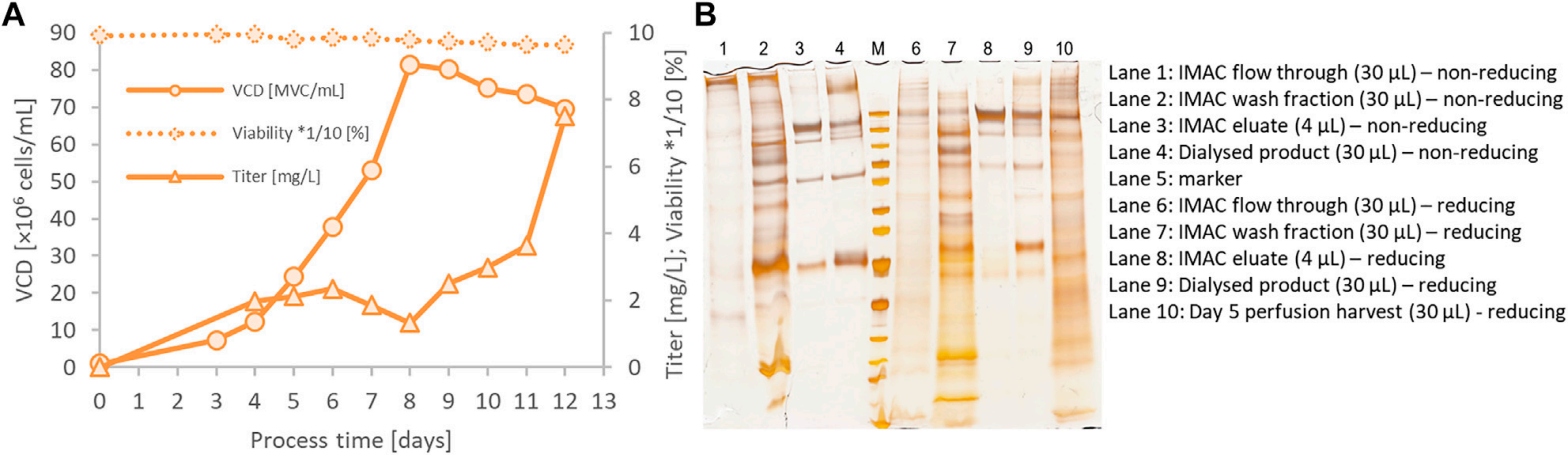
500 ml bioreactor perfusion run for integrated bioprocessing of the CSs spike protein using a preliminary low-producing subclone (CHO-K1 CSs 131A4) **(A)**. The bioreactor harvest was coupled directly to an Äkta Pure system equipped with a HisTrap Excel column. On day 8, cell bleeding was started to keep viable cell density (VCD) below 80 × 10^6^ viable cells/ml. IMAC fractions, dialyzed end product, and perfusion harvest on day 5 were analyzed by silver-stained SDS-PAGE **(B)**.

The parental CSs clone 131A4 was used in an integrated 500 ml perfusion bioreactor directly connected to the Äkta Pure system equipped with a HisTrap Excel column (Figure 4) to generate large harvest volumes compensating for the relatively low product titer. The perfusion rate of 0.2 vessel volumes per day (vvd) was kept constant from days 3 to 8 followed by a stepwise increase to 2 vvd on day 11 to prevent nutrient limitation. In the steady-state of the perfusion, the viable cell density (VCD) reached 80 × 10^6^ viable cells/ml, maintained by a continuous cell bleed starting on day 8 (Figure 4A). This adjustment translates into optimized cell-specific perfusion rates between 10 and 40 pl medium per cell per day (pL/c/d) for minimized medium consumption and increased product titer. Integrated direct purification was initiated on day 5. The continuous perfusion harvest was mixed continuously with 50% (v/v) of phosphate buffer and loaded onto a HisTrap Excel column. This column minimizes metal stripping off the column caused by extended exposure to the cell culture medium. The first IMAC elution fraction was collected the day after loading and dialyzed against 20 mM Tris-HCl, 150 mM NaCl, pH 8.0. High-molecular weight species were visible in silver-stained SDS-PAGE (Figure 4B) that were enriched in the dialyzed product (lane 4) compared with the IMAC elution fraction (lane 3). These aggregates were partly resolved under reducing conditions (lane 9). Inefficient binding of the CSs protein led to significant amounts of CSs protein in the flow-through fraction (FT, lane 1 and 6) and wash fraction (WS2, lane 2 and 7). Thus, although the perfusion process in the bioreactor was successful and high performing concerning the cell culture parameters, further optimization would be required for the integrated downstream purification process for optimized yield and purity in the eluate fraction.

**FIGURE 5:**
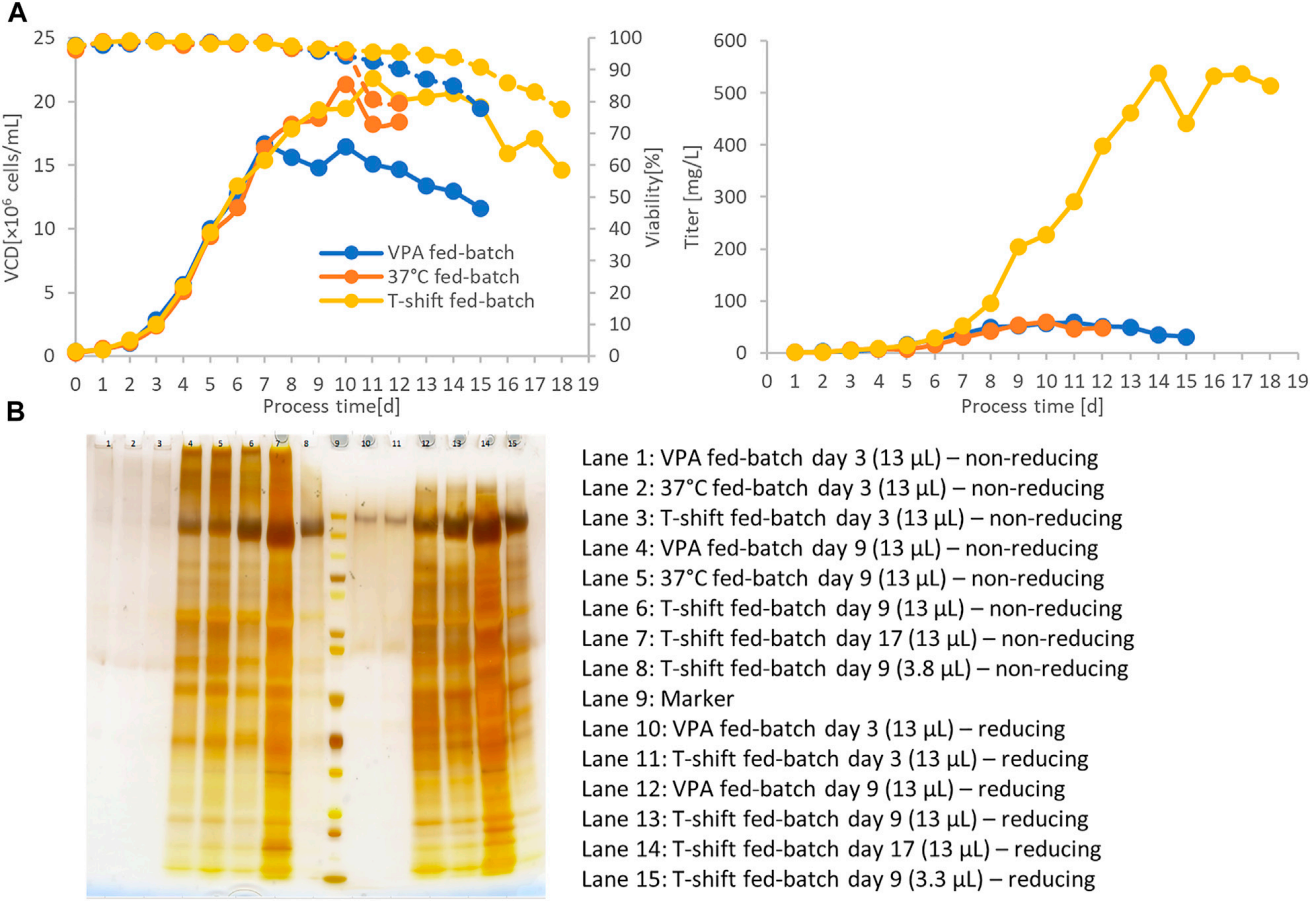
500-ml fed-batch bioreactor cultivation of the final C11 clone at 37°C, hypothermic temperature shift to 32°C or valproic acid (VPA) addition **(A)**. Culture longevity and final harvest titer were significantly improved by shifting to 32°C after day 6. Titer was measured by a quantitative ACE2/anti-His-btn ELISA setup. SDS-PAGE also verified the significantly higher expression titer of hypothermic fed-batch cultivation by an equivolumetric comparison **(B)**. Sample volume of 13 µl showed the enriched product band in the 32°C culture supernatant.

In another experiment, the final CSs clone C11 was cultivated in lab-scale fed-batch bioreactors. Various fed-batch strategies were tested to boost final spike protein titers in the culture supernatant. Valproic acid (VPA) is a small molecule compound that improves protein expression in mammalian cells, acting as an inhibitor of histone deacetylases (iHDAC). Furthermore, hypothermic cultivation temperatures limit cellular growth but increase cell-specific recombinant protein expression. In transient spike protein expression, a beneficial effect of lowered culture temperatures was demonstrated that led to titer enhancements of more than 10-fold in previous studies (Esposito et al., 2020). In another study, hypothermic conditions and VPA addition increased product titers by a factor of five in stable CHO pools (Johari et al., 2021). Using the selected production clone C11 in bioreactor fed-batch cultivation, we observed that viability dropped to 80% on day 11 at standard 37°C cultivation temperature (Figure 5A). Culture longevity was induced by a hypothermic temperature shift to 32°C after day 6 or a single shot of valproic acid on day 6. A maximum VCD of 20 × 10^6^ viable cells/ml was reached for both the standard 37°C and hypothermic 32°C cultures, but only 15 × 10^6^ viable cells/ml for the culture supplemented with VPA. Hypothermic conditions prolonged process duration up to 18 days at viabilities above 80%. Despite the induction of higher cell-specific productivities, VPA addition was not suitable to obtain higher CSs titers because of reduced VCD.

Interestingly, hypothermic treatment has induced exceptionally high product titers of up to 500 mg/L. Despite similar peak VCD, the temperature shift has induced a harvest titer 10-fold higher than the default fed-batch process of 37°C. In order to eliminate the possibility of artifacts in the ELISA quantification due to misfolded CSs from the hypothermic condition, equal supernatant volumes from the different fed-batch conditions were applied on an SDS-PAGE for selected culture days (days 3 and 9). An enriched product band confirmed higher product titers under the hypothermic fed-batch condition (Figure 5B, lane 7 and 14).

**FIGURE 6:**
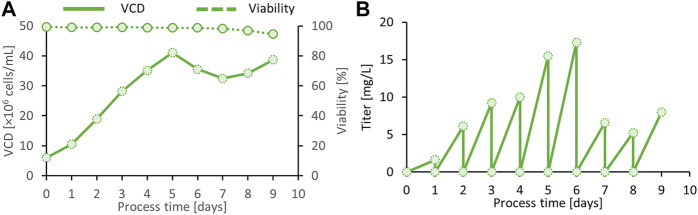
Viable cell density (VCD) and viability of the final clone C11 producing the SARS CoV-2 spike protein in semi-continuous perfusion mode **(A)**. Daily harvest titer was measured by a quantitative ACE-2/anti-His sandwich ELISA **(B)**.

Alternatively, we developed a small-scale semi-continuous perfusion culture that keeps cells in a healthy state for prolonged periods at exceptionally high cell densities of 40 × 10^6^ viable cells/ml in uncontrolled 200-ml shake tubes. This procedure follows a daily medium exchange of one reactor volume per day and provides daily supernatant harvests of up to 15 mg/L spike protein (Figure 6). Compared with the fed-batch process, cells at higher VCD are kept at higher viabilities, and consequently, the best product quality can be expected.

### Optimized Immobilized Metal Affinity Chromatography for Highly Pure and Functional Trimeric Spike Protein Preparation

**FIGURE 7:**
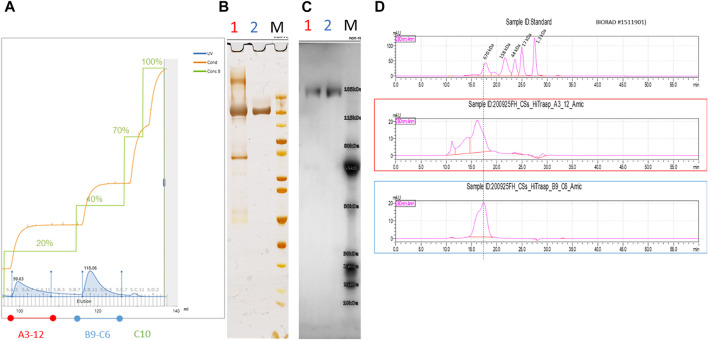
IMAC purification of spike protein using a 20%–40%–70%–100% step gradient protocol **(A)**. Spike protein eluted at 40% (B9-C6) provided highly pure and functional fractions verified by non-reducing SDS-PAGE/Silver staining **(B)** and Western blotting against hyperimmune IgG **(C)**. A correct band size of approximately 180 kDa was obtained, which would be expected for a denatured monomeric CSs protein chain. Polyclonal antibodies against the spike protein were isolated from hyperimmune sera by cryo-and ammonium sulfate precipitation, followed by protein A affinity chromatography and biotinylation for Western blotting. Analytical SEC analysis confirmed the high purity of fraction B9-C6 (blue) with only the characteristic peak visible **(D)**.

Immobilized metal affinity chromatography (IMAC) was chosen as the preferred purification method of the CSs protein by using a chelating HisTrap column loaded with Ni-ions and a stepwise increased imidazole gradient. In the first experiment, elution buffer was increased in 5% increments and fractions analyzed by ELISA. In fractions with low imidazole concentration, the bound spike protein coelutes with host-cell proteins, whereas, at higher imidazole concentrations, the spike protein can be isolated in a pure form. Consequently, a stepwise elution profile at 20%, 40%, 70%, and 100% of 500 mM imidazole concentration was defined in 20 mM phosphate buffer with 150 mM NaCl, pH 7.4 and was used to purify the culture supernatant from the semi-perfusion process (Figure 7A). Different HisTrap elution fractions were analyzed by non-reducing SDS-PAGE/silver stain (Figure 7B), Western blotting with hyperimmune IgG (Figure 7C), and SEC-HPLC (Figure 7D **)**. This confirmed that CSs protein can be isolated with high purity from the 40% gradient step (fraction B9–C6) (Figure 7A).

IMAC purification shows several disadvantages concerning low binding affinity (Riguero et al., 2020), coelution of host-cell proteins, ion stripping, and incompatibility of cell culture media components (Farnós et al., 2020; Johari et al., 2021). Thus, many laboratories later switched to alternative affinity chromatography resin (e.g., Strep-Tactin). Although we developed a suitable purification protocol for highly pure spike protein, a significant fraction of the expressed protein was lost in the first wash step. Furthermore, significant amounts of CSs protein were detected in the flow-through. This was also observed by Stuible et al. (2021) for a Ni-Sepharose excel resin and attributed the reduced binding capacity to the size and/or shape of the spike construct, which may prevent diffusion into the pores. Further optimization of the IMAC procedure would be required to improve the final yield by optimizing the step gradient, choosing a gradient elution profile, or alternative purification methods (Cibelli et al., 2021).

**FIGURE 8:**
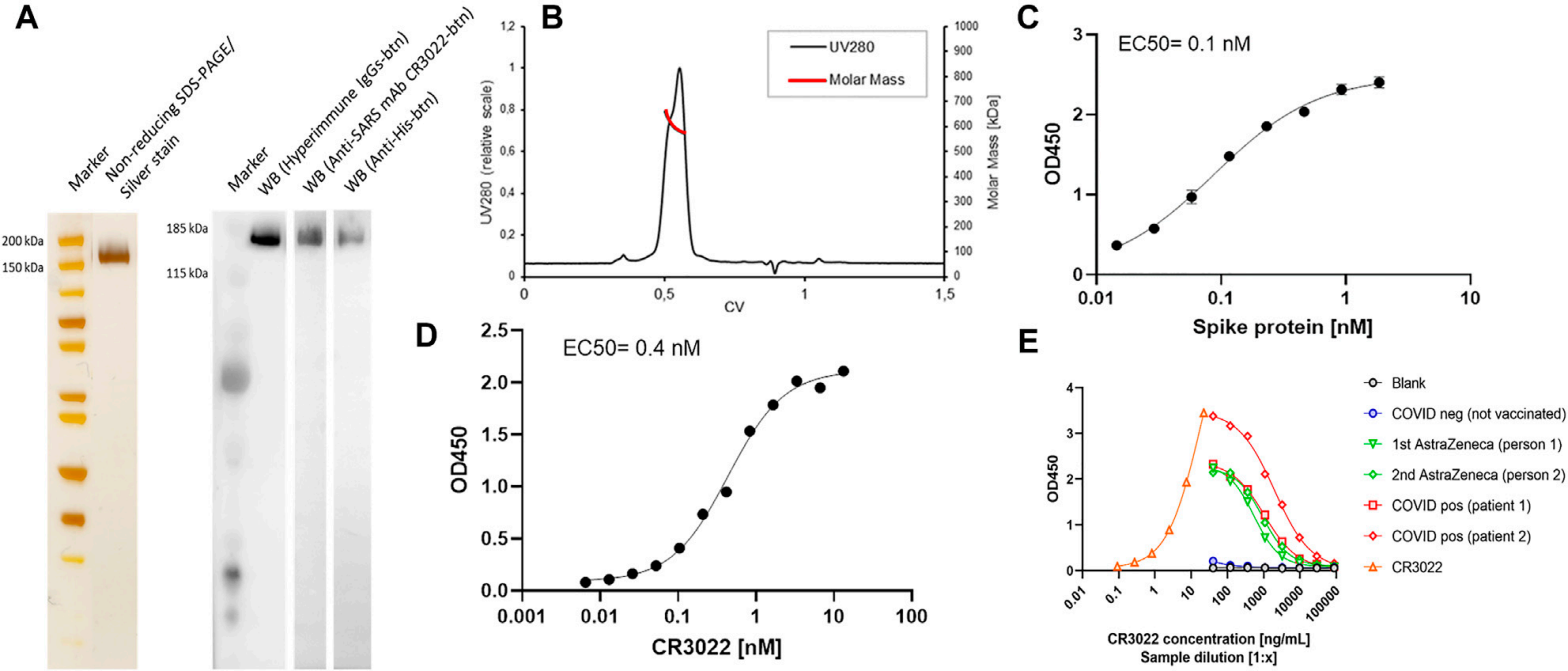
Non-reducing SDS-PAGE of the pure CSs spike protein preparation gives a single intense band (2 µg loaded) after silver stain and various Western blotting setups using hyperimmune IgG, anti-SARS mAb CR3022, or anti-His antibodies **(A)**. Analytical SEC/MALS-HPLC by a Superose 6 increase 10/300 GL column (Cytiva) indicates the high purity of the IMAC preparation with the characteristic double peak of the spike protein **(B)**. The molar mass of the trimeric spike is about 610 kDa (MALS signal) and elutes at 0.55 CV. The purity of the trimer fraction is >90%. Determination of affinity (EC50) of spike protein to ACE2 **(C)**: ACE2 was coated on ELISA plates, and the bound CSs spike protein was detected by anti-His-btn/SA-HRP conjugates. The purified spike protein preparation showed high affinity to the anti-SARS mAb CR3022 **(D)**: Coating: 5 μg/ml spike protein and detection with CR3022-btn/SA-HRP conjugates. A sigmoidal non-linear four-parametric log model was used for the calculation of the EC50. The purified spike protein was applied to a serological ELISA set up to detect spike-specific antibodies in COVID-positive individuals or individuals vaccinated with one or two doses of the AstraZeneca vaccine **(E)**: coating 5 μg/ml of spike protein and detection with anti-huGamma-HRP.

**FIGURE 9:**
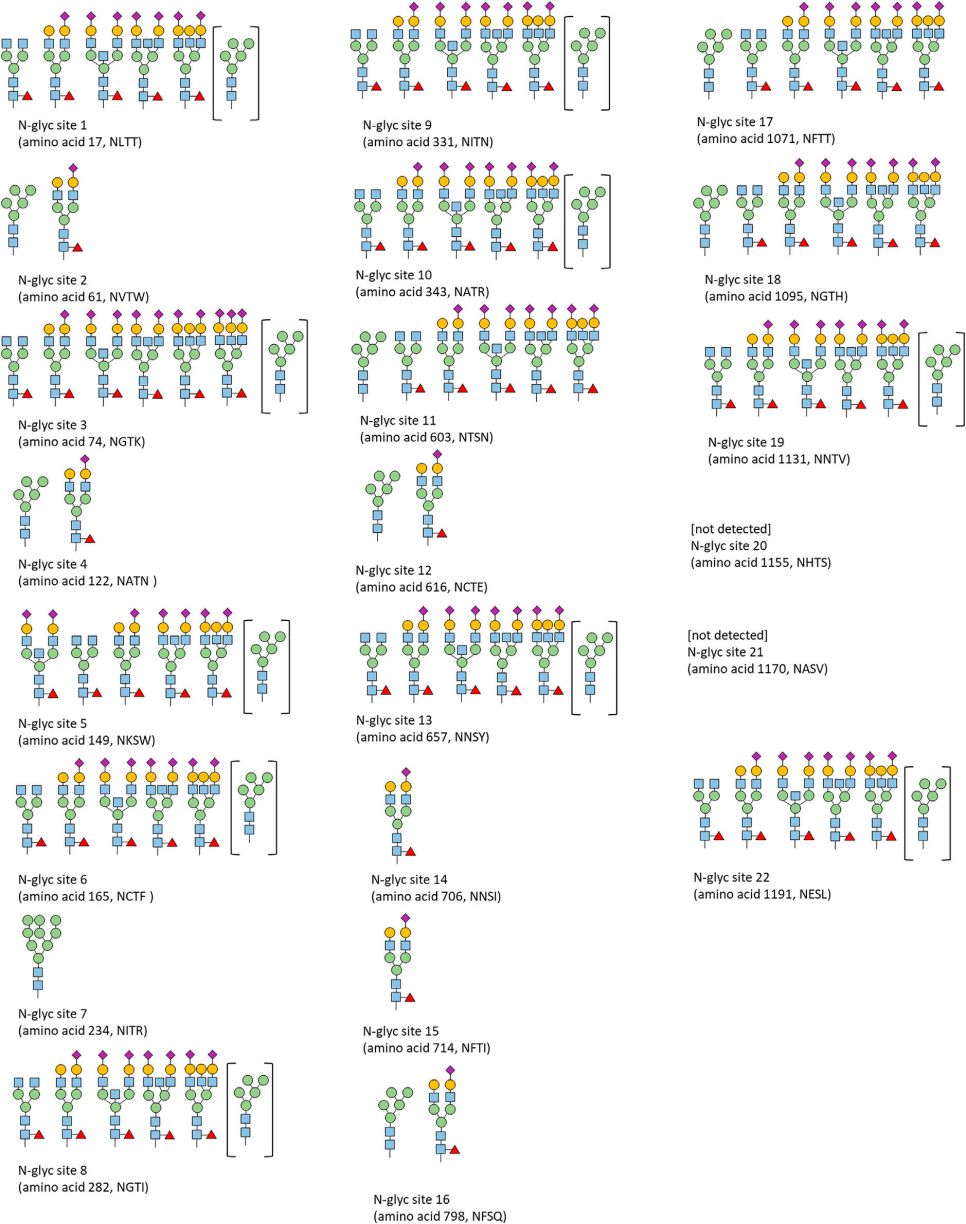
Qualitative N-glycosylation analysis of CSs spike protein by LC-ESI-MS/MS.

The IMAC purified CSs protein was characterized in more detail by SDS-PAGE, Western blotting, ELISA, and N-glycan analysis (Figure 8 and Figure 9). A single defined band with the correct size of the CSs monomeric chain was observed in non-reducing SDS-PAGE and Western blotting using biotinylated hyperimmune IgG, CR3022, or anti-His mAbs (Figure 8A). The SEC profile showed the characteristic double peak with the correct molar mass of a fully glycosylated and trimeric CSs protein (Figure 8B). EC50 against ACE2 or CR3022 was determined at 0.1 and 0.4 nM, respectively (Figures 8C, D) and agrees with previously published literature (Tian et al., 2020; Walls et al., 2020). The purified CSs spike protein was then successfully applied in serological ELISAs against COVID negative and positive sera or vaccinated individuals. The produced spike protein generated a strong response for binding of anti-SARS mAb CR3022 and anti-SARS-CoV-2 hyperimmune IgG present in convalescent sera of COVID patients or vaccinees after one or two doses of the AstraZeneca vaccine (Figure 8E).

N-glycosylation was qualitatively analyzed by LC-ESI-MS/MS analysis (Figure 9). The identified glycosylation patterns are in agreement with previously published glycan patterns of the spike protein. Specifically, for sites 2, 4, 7, 11, 16, and 17, a significant fraction of oligomannose type was reported in the literature (Watanabe et al., 2020; Allen et al., 2021; Hoffmann et al., 2021).

## Conclusion

Efficient spike protein expression is crucial for biomedical research, subunit vaccine production, and the development of serological tests to screen the success of vaccines and evaluate the immunological status of previous infections. Thus, at the pandemic onset, the goal was to rapidly produce the novel spike protein in large quantities at the highest quality. It is evident that transient transfection has established itself as the preferred method to produce low amounts rapidly but with significant resources and costs at small-scale. Literature on stable cell lines expressing the viral spike protein for large-scale production is scarce due to the complexity and timely process of generating clonally derived, high-producing cell lines. Compared with 19 transient approaches only three publications with stable cell lines were found in the rather extensive literature search (Allen et al., 2021; Cibelli et al., 2021; Johari et al., 2021). We demonstrate how the established technological repertoire in mammalian cell expression can be efficiently adapted to express the trimeric spike protein in large amounts in stable clonally derived CHO cell lines. The CHO-K1 host cell line previously showed beneficial capabilities for growth and productivity compared with other host cell lines (Reinhart et al., 2018) and was used to generate recombinant CSs producing subclones. Furthermore, the CHO-K1 host shows beneficial glycosylation capabilities and is safe regarding the risk of contamination and transmission of human pathogens. Low but sufficient amounts of spike protein were initially produced with an early transfection pool by a small-scale semi-continuous or lab-scale bioreactor perfusion process. During the clone development workflow, it is crucial to generate and provide spike protein from low-producing cell pools to develop specific analytical and downstream purification methods. These methods enabled us to establish high-producing, clonally derived cell lines, which were then used to develop a fed-batch process at 20 × 10^6^ cells/ml.

Interestingly, a hypothermic fed-batch temperature shift to 32°C induced a 10-fold increase in the final harvest titer. As Johari et al. (2021) pointed out, improved purification methods are required to cope with host cell impurities at high cell densities. Thus, we also developed a continuous bioreactor perfusion process at 80 × 10^6^ cells/ml or semi-continuous shake tube bioprocesses operated at the highest cell viabilities above 95% over a prolonged time. While the presented IMAC purification step gradient provided a sufficient amount of highly pure CSs protein from highly productive cell cultures, it was not further optimized to provide the highest yields due to co-elution with host cell proteins and product loss at low imidazole concentrations. Recently, an optimized IMAC method or alternative capture methods were demonstrated for SARS-CoV-2 spike proteins (Cibelli et al., 2021; Johari et al., 2021). The development of robust protocols finally enabled assessing the quality of the expressed spike protein by various setups for Western blotting and ELISA. The purified spike protein from stable high-producing CHO clones showed the correct band size in SDS-PAGE and Western blotting using anti-His, the anti-SARS mAb CR3022, and polyclonal antibodies isolated from convalescent plasma. Analytical SEC-MALS showed high purity of the spike protein preparation from the optimized IMAC protocol. However, the purification protocol was optimized for purity and quality that came with costs for product yield. The purified spike protein showed high affinity to the ACE2 receptor and the anti-SARS mAb CR3022 in the established ELISA. Finally, the purified spike protein was successfully applied in a serological ELISA setup to screen COVID-positive sera and two of vaccinated individuals.

In summary, this study demonstrates the optimal combination of CHO-K1 host cell line, expression vector, and fed-batch or continuous bioprocesses to produce the complex spike protein in large amounts at the highest quality and is already used successfully in ongoing serological studies or to develop novel anti-SARS-CoV-2 antibody formats (Garner-Spitzer et al., 2021; Kallolimath et al., 2021; Sun et al., 2021). The availability of viral proteins in high quality and sufficient amounts now allows investigating the biological and virologic basics of SARS-CoV-2 to understand the infection and propagation mechanisms, the virus distribution in the body, the docking of the virus, viral immune evasion, and the immune response mechanism of the human body in more detail. At an unprecedented speed, various laboratories worldwide are now focusing on this emerging pathogen and combining their expertise to investigate the new spike protein of this virus to reach one common goal: Understand the virus, and exploit its weaknesses for effective treatments and vaccines. The shared lessons from this pandemic will prepare us for future challenges.

## Data Availability

The original contributions presented in the study are included in the article. Further inquiries can be directed to the corresponding author.
